# Supplementary material for: The relation between local and distal muscle fat infiltration in chronic whiplash using magnetic resonance imaging
Source: PLoS One. 2019 Dec 5;14(12):e0226037. doi: 10.1371/journal.pone.0226037 (PMC6894804; doi:10.1371/journal.pone.0226037)
Supplement: Attachment — Submitted filename: ResponsetoReviewers.docx [file pone.0226037.s002.docx]

**Reviewer #1:**
**General Comments:**

**I appreciate the substantial amount of time and effort that had to have gone into this study. I hope my comments on clarifying and narrowing the study focus and physiological premise/framework will be helpful to improve the manuscript.**

*Thank you for being part of the review process. We believe that the changes made from your valuable comments have significantly improved the quality of the manuscript.*

**The study purpose and scientific premise of the study are unclear. At times, the manuscript feels like a technical paper describing an improvement on prior MRI imaging techniques used in individuals with whiplash. Other times, it feels like the manuscript is asking a more clinical or mechanistic question.**

*In order to clarify the paper’s premise and purpose we have discarded the purpose related to the choice of MR imaging technique and therefore solely focusing on the clinical and mechanistical aims.*

**There needs to be stronger support for the scientific premise of comparing multifidus fatty infiltration to whole lower limb infiltration in this population. It seems factors like obesity, sedentary behavior, or diabetes that are unaccounted for in this study would cause some individuals to have the tendency toward fatty infiltration throughout the entire body irrespective of whether or not they’ve sustained a whiplash injury. The rationale for why this relationship is important to quantify in the context of whiplash is unclear to me. The introduction needs to more clearly set up the importance and scientific premise of the research question.**

*We have provided some additional support from literature why comparing multifidus fatty infiltration to whole lower limb fatty infiltration is of interest in this population. We moved the first paragraph on p 12 from the discussion to the introduction as suggested and cleared up the aims of the study. We believe that the introduction now better explains the premises of this study.*

**Specific Comments:

Introduction
- 2nd paragraph, sentence beginning “Refined and extended measurements…” - Need to support this statement. There are other means to investigate the mechanisms that you list prior to this statement. What mechanism are you testing by imaging the lower limbs and the multifidus?**

*In the new version of the introduction the sentence has been replaced. The new version of the introduction explains the potential injury in the cervical spine as one hypothesis for the potential pathophysiological link between local and distal MFI in the WAD cohort.*

**- The multifidus is only mentioned late in the methods section. Recommend addressing in introduction why this muscle is so important to study rather than studying deep neck flexors, for example.**

*We have put more emphasis on the motivation for studying only multifidi (in contrast to all deep neck flexors) already in the introduction.*

**Methods
- Were there significant differences in age or time since injury between WAD groups? Is there concern that differences (particularly in time from injury) could influence the results?**

*We have added a regression analysis between MFI in multifidi against duration since injury in the WAD groups. They were highly uncorrelated and we added that in the manuscript. Hence, we don’t think duration would influence the result at all. Age and MFI are often associated and so also in this study. Therefore, we have adjusted for age in the model but local and distal MFI are still associated which shows that the association remains with age taken into consideration.*

**- Were participants excluded from the healthy group if they had a history of lower extremity injury? Were participants excluded from the injured groups if they had a history of lower extremity injury (associated or not associated with accident/onset of whiplash) in the absence of current pain complaints?**

*Neither the healthy group nor the WAD groups were investigated whether they had a history of lower extremity injury or not. However, an exclusion criterion for the study was generalized or dominant pain in other parts of the body. Furthermore, none of the participants had implants in the lower extremities. We added this to the limitation in the discussion.*

**- P.7: Clarify the credentials of the medical imaging expert (Physician? Researcher? Radiologist?)**

*This has now been clarified.*

**- In introduction of methods, be explicit about how the MRI technique used in this study is different from previously used techniques**

*This has now been clarified both in the introduction and in the method section.*

**- Was volume of multifi normalized to participant height or number of slices? Is it possible that the size of the person is a confounder?**

*We have not been investigating the volume of multifidi but the fat infiltration. Since fat infiltration is only a fraction of the total volume, the size of the muscle/person should not be a confounder. However, it is a reasonable hypothesis that BMI can affect the MFI, and we added that as a factor in the regression model.*

**Results
- I am not seeing any results comparing the MRI technique used in this study with the 4-slice technique mentioned**

*The aim of comparing the methods has been discarded from this version of the manuscript and the figure related to those results has also been discarded.*

**Discussion
- As you clarify the focus of this manuscript and the mechanism being investigated, there are likely going to be substantial revisions to this section to align it with the study purpose.**

We believe that the revision of the entire paper also has satisfied this comment.

**- P. 11: “indicate a lower variance of MFI within the groups.” P-values do not quantitatively indicate variance between groups. Perhaps, coefficient of variation? The remainder of this paragraph refers to data that is not in the results section. (Referenced fig. 3 in 3rd paragraph on p. 11 does not show the data suggested by the text.)**

*We agree with the arguing above. Since we have removed the aim of investigating the different imaging techniques, the paragraph mentioned above has also been removed.*

**- P. 12: I think the first paragraph on this page is really starting to get at your overarching physiological premise. I’d recommend moving all or parts of this paragraph to the introduction and expanding this section to be very clear to the reader what you are investigating.**

*We have moved the paragraph to the introduction, which should clarify the physiological premise of this study.*

**- P. 13: I encourage you to consider the possibility that relationships in fatty infiltrate throughout the body are probably very reasonable (and based on your data do not to be associated with injury). There were differences between groups in multifidus infiltrate, so it seems like limiting imaging to the cervical spine would be reasonable in this patient group.**

*Yes, we agree that an overall relationship between the different groups and whole body is very reasonable. We have extended the linear regression in this study by expanding the model looking at each group (severe WAD, mild/moderate WAD and healthy controls). This analysis actually showed a much stronger relationship for the severe WAD group, which has now been added to the results.*

**Reviewer #2:**

**General Comments:

The objective of this study was two-fold: one it seeks to determine whether fat infiltration (MFI) in the cervical muscles following whiplash injury is associated with distal muscle MFI and two, it examines the benefits of using a comprehensive 3D measurement of MFI over selective cross-sectional slices. These are disparate examinations and one does not seem to meaningfully link to the other. Though data are presented toward both, neither is tested or discussed in sufficient detail to draw compelling conclusions. Thus, it is recommended that these individual investigations be split into two manuscripts and fleshed out so as to contribute meaningfully to the literature. Specific recommendations on how to do this are provided below.**

*We thank the reviewer for the time to provide suggestions for improving our manuscript. We have discarded the objective of investigation regarding the benefits of a comprehensive 3D measurement of MFI and focus the paper of the local and distal fat infiltration.*

**Specific Comments:
1. The motivation for investigating the link between cervical and lower extremity MFI is unclear as is the interpretation of results. Why would you expect these to be linked with injury? Do individuals with chronic WAD frequently report lower body symptoms? An MRI with whole body coverage adds significant time and expense and is unlikely to be implemented to “inform assessment and management” unless it offers improved diagnostic power. It will be necessary to present a hypothesis based on published data or mechanism to justify the investigation on these grounds.**

*We have provided some additional support from literature why comparing multifidus fatty infiltration to whole lower limb fatty infiltration is of interest in this population. We moved a paragraph from the discussion to the introduction and cleared up the aims of the study. We believe that the introduction now better explains the premises of this study.*

**On the other hand, I think the more interesting take on this investigation is the fact that cervical MFI and persistent symptoms might be linked for other reasons. For instance, increased age, BMI and prevalence of co-morbidities such as diabetes might predispose one to have increased total body MFI and poor recovery from whiplash injury. Alternatively, those with persistent symptoms may have adopted a sedentary lifestyle leading to increased MFI.**

*In order to further evaluate potential confounders, we have clarified potential confounders in the method section. We also added BMI in the linear regression analysis and also did a regression between MFI in multifidi and duration since injury in the WAD groups.*

**2. The MFI quantification techniques were different between the cervical muscles and the lower extremity. Lower resolution and complete automation of the segmentation likely decreased precision in the measurement of lower extremity MFI, which could be why significance was not obtained. Of course, the MRI sequences cannot be adjusted now, but can the same segmentation strategy be used between regions?**

*Since the multifidi is such a small muscle it is quite challenging acquiring data with high enough resolution in an MRI scanner in order to be able to quantify the fat content. Therefore, that method is not as stable as the lower extremity method. To clarify this in the manuscript we present the differences between the methods in the method section. We also added the difference between methodologies as a limitation in the discussion.*

**3. Why was cervical-lower extremity MFI regression analysis run only on the complete cohort of 62 participants? Wouldn’t you expect there to be a different relationship between these variables in control vs. severe WAD groups? Along those lines, there are several outliers in the regression. To which group do these participants belong?**

*We have now extended the regression analysis to be run on the different WAD groups and controls alone. This analysis actually showed a much stronger relationship for the severe WAD group, which has now been added to the results. See figure 3 in the new version of the manuscript.*

**4. While age was considered as a confounder, other variables independently associated with MFI were not. If the data is available, BMI and diagnosis of diabetes should be evaluated. Are any evaluations of lower extremity function performed on these individuals as part of the larger study? Could this correlate with the larger MFI in the severe WAD group?**

*No participant had diabetes as it was an exclusion criterion in the study. BMI was added to the model. We have not done lower extremity functional tests and have added that as a limitation in the discussion section.*

**5. The argument for making whole muscle measurements of MFI vs. single or several slice measurements is (I assume) that MFI is spatially heterogeneous and thus a single slice could suffer from selection bias. Please add your findings on the spatial distribution of MFI in the cervical muscles. Is it consistently higher in certain muscle regions? Does its distribution change with injury?**

*We removed the aim related to this question. However, we do know there are spatial variabilities. Both reference 5 and reference 6 in the manuscript report differences in fat infiltration along the different discs C4-C7.*

**6. Were the same segmentation methods used for the 2-D images? It is surprising that the values obtained in the 3D analysis are half to a third of what they are in the 2-D analysis. No conclusion can be drawn as to the benefit of a 3D analysis in reducing variance if the segmentation strategy is different.**

*No, the segmentation methods were slightly different. We agree that such a conclusion is not suitable to be drawn when the methods are different. We have removed that aim from this study.*

**7. Cervical muscle cross-sectional area changes following whiplash are also somewhat contentious. Is there bias in estimating the cross-sectional area from a single slice? How variable is this measure along the length of the muscle?**

*Yes, that is true that cross-sectional area is contentious. In this study we only consider the fraction of fat in multifidi and do not look at cross-sectional area nor volume. Since we now only measure the fat fraction of the whole volume in both lower extremities and multifidi in this manuscript we are not sensitive to slice positioning.*
