# Supplementary material for: The relation between local and distal muscle fat infiltration in chronic whiplash using magnetic resonance imaging
Source: PLoS One. 2019 Dec 5;14(12):e0226037. doi: 10.1371/journal.pone.0226037 (PMC6894804; doi:10.1371/journal.pone.0226037)
Supplement: Attachment — Submitted filename: Plos1_Review_Karlsson.docx [file pone.0226037.s003.docx]

Review – Manuscript Number: PONE-D-19-19642

The relation between local and distal muscle fat infiltration in chronic whiplash using magnetic resonance imaging

General Comments:

I thank the authors for their work in addressing my comments and believe the paper is now much more focused and streamlined. I have a few additional comments and suggestions primarily related to the discussion of the data and conclusions drawn.

Specific Comments:

1. Introduction: I am still struggling with the connection between this data and the idea of “complementing the neck images with whole body coverage” for diagnostic purposes. First, you are unable to determine whether the stronger correlation between multifidi and lower extremity MFI in the severe WAD group pre-exists and predicts a worse outcome or is a progressive response to injury. The data that you discuss more strongly support the latter, in which case diagnostic whole body scans would not be useful. Second, to argue for whole body scans, you would have to show that the relationship between multifidi and lower extremity MFI is a better indicator than multifidi MFI on its own. This can be grossly assessed by adding the two tests discussed in the next points.

2. Results: Please perform an ANCOVA on your three regressions to determine whether the slopes are significantly different.

3. Results: Please also include an ANOVA on the multifidi MFI between groups to complement Figure 4.

4. Results: Please report all statistical results in your Results section. The Discussion discusses associations between multifidi MFI and age, BMI and duration since injury and a comparison of multifidi MFI across groups. If these are outcomes of the statistical models, please include more details about the models and their outputs so the reader is aware of the results before the discussion.

5. Discussion: Please discuss additional explanations for the association between multifidi and lower extremity MFI. These are mentioned in the Introduction, but I would like to see a more in-depth treatment in the Discussion. “However, a number of hypotheses around injury severity, pain intensity and/or related disability (9, 10), heightened stress-responses (11-13) central/peripheral neuronal interference (14-16), and/or physical inactivity (17, 18) may be offered to explain the rapid expression and larger magnitude of MFI in patients with more severe self-reported symptoms.”
